# Bipolar disorder and socioeconomic status: what is the nature of this relationship?

**DOI:** 10.1186/2194-7511-1-9

**Published:** 2013-06-21

**Authors:** Laeticia Eid, Katrina Heim, Sarah Doucette, Shannon McCloskey, Anne Duffy, Paul Grof

**Affiliations:** School of Medicine, Queen’s University, Kingston, Ontario K7L 3N6 Canada; Mood Disorders Centre of Ottawa, Ottawa, Ontario K1G 4G3 Canada; Department of Community Health and Epidemiology, Dalhousie University, Halifax, Nova Scotia B3H 4R2 Canada; Department of Psychiatry, University of Calgary, Calgary, Alberta T2N 1N4 Canada; Department of Psychiatry, University of Toronto, Toronto, Ontario M5S 1A1 Canada

**Keywords:** Bipolar disorder, Lithium response, Socioeconomic status, Longitudinal studies, Gender

## Abstract

**Background:**

In psychiatric literature stretching over a century, there have been glaring discrepancies in the findings describing the relationship between bipolar disorder (BD) and socioeconomic status (SES). Early studies indicated an overall association between manic-depressive illness and higher social class. However, recent epidemiologic studies have failed to find an association between BD and SES. Instead, they report a similar distribution of BD among social classes and educational levels, and in one particular study, a lower family income was reported. The determinants of SES are complex, and the early findings are now interpreted as having been incorrect and stemming from past methodological weaknesses.

**Methods:**

For this analysis we explored the relationship between SES and BD in a sample of patients who had participated in prior clinical and therapeutic studies. These patients met the *Diagnostic and Statistical Manual of Mental Disorders, Fourth Edition* criteria for BD, required long-term stabilizing treatment, and were assessed in terms of their response to lithium stabilization and a number of other clinical characteristics in accordance with research protocol. Good response to lithium stabilization (LiR) served as a proxy for identifying a subtype of manic-depressive illness, the classical form of BD. Non-responders to stabilizing lithium (LiNR) were considered belonging to other subtypes of bipolar spectrum disorder. The SES of the parents was measured upon entry into treatment using the Hollingshead SES scale, which despite its limitations has been used in psychiatry most widely to determine SES. The groups of LiR and LiNR were compared statistically in terms of SES. The influence of bipolar subtype and gender on SES was investigated.

**Results and discussion:**

*A significantly higher SES was associated with the lithium-responsive form (LiR) of BD* when compared with patients continuing to relapse despite adequate lithium treatment (representing other types of bipolar spectrum). Our observation suggests that the discrepant literature findings about SES and BD may be better explained by the change in diagnostic practices: early studies describing a positive relationship included mostly classical manic-depressive disorder, while the patients in recent studies have been diagnosed according to much broader criteria, reflecting the era of bipolar spectrum disorder.

## Background

The relationship between socioeconomic status (SES) and mental health is not only interesting but also important when considering determinants of health, access to care, treatment compliance, and prognosis. Bipolar disorder (BD) is no exception. It is a life-long illness characterized by recurrences of mania and depression, which can cause impairments in functioning and health-related quality of life (Rosa et al. 2008). The mood symptoms associated with BD can cause clinically significant distress or impairment in many different areas of a person's life, including but not limited to social and occupational areas of functioning.

Over the years, there have been glaring discrepancies in the findings describing the relationship between BD and SES. Over 40 studies have been reported, most of them reviewed in the comprehensive textbook of Goodwin and Jamison (1990). In the older studies, it was usually noted that the average SES of those with BD was relatively higher than that of controls or the general population (e.g., Malzberg 1956;Verdoux and Bourgeois 1995). However, many recent epidemiologic studies have failed to find such differences. They report a similar distribution of BD among educational levels (e.g., Schoeyen et al. 2011), and in recent literature even a lower social status was reported (Schoeyen et al. 2011;Tsuchiya et al. 2004).

For example, in an early study reported by Coryell et al. (1989), probands were diagnosed according to Research Diagnostic Criteria. The sample consisted of 422 probands with major depression and 152 with Bipolar Disorder I (BDI) and Bipolar Disorder II (BDII). The researchers used the Hollingshead criteria to ascertain education and occupation and ultimately approximate SES. The results of this study indicated that first-degree relatives of BD probands had significantly higher educational and occupational levels than those with major depression. Similarly, Lenzi et al. (1993) conducted a study with analogous methodology on earlier data. Experienced psychiatrists analyzed the data on 877 patients who took part in psychopharmacological trials between the years of 1975 and 1982, and made diagnoses according to the DSM-III-R. SES was measured using the Hollingshead criteria, and again, the results showed that the upper social classes had a higher proportion of BD patients than did the lower social classes. These results were observed even with the use of different methodologies (Goodwin and Jamison 1990;Tietze et al. 1941). Similarly, studies conducted in various countries such as Germany (Luxenburger 1933), Norway (Noreik and Odegaard 1966), India (Rao 1966), Israel (Gershon and Liebowitz 1975), and Sweden (Petterson 1977) all observed the same trend.

Later studies used similar assessments, but different methodological elements for calculating SES than those referenced above, and had strikingly different results. For instance, a recent study conducted by Goldstein et al. (2010) sampled 288 offspring of parents with BD diagnosed using the *Diagnostic and Statistical Manual of Mental Disorders, Fourth Edition* (DSM-IV) criteria and again calculated SES using the Hollingshead criteria. This study found that offspring with BD had parents with a significantly *lower* SES than the reference sample (Goldstein et al. 2010). However, the possible explanation for this finding may be that the US samples of high-risk offspring usually report many comorbid diagnoses and recruit directly from the general population. Schoeyen et al. (2011) sampled 257 Norwegian patients with BD (diagnosed using DSM-IV criteria) and a reference group consisting of 56,540 people from the general population. SES was calculated by considering education and income separately. Like in the study by Goldstein et al. (2010), those with BD had a lower income than the reference population, although no difference in educational level was noted (Schoeyen et al. 2011).

Goodwin and Jamison (1990) pointed out that earlier studies are difficult to interpret due to a wide variation in the criteria used to define social class between studies. Furthermore, it has been suggested that the early findings were inaccurate and stem from methodological deficiencies such as treatment bias, lack of appropriate reference samples, and failure to control for demographic variables (Schoeyen et al. 2011).

It is important to explore the possible explanations of this discrepancy in the literature since SES is very relevant in the discussion of etiology, social systems, and treatment compliance (Coryell et al. 1989). Although methodological differences and weaknesses in earlier studies may in part explain the differences in findings regarding SES and BD, there are a number of other important clinical differences to consider. Many of the earlier studies examined samples of patients with a classical presentation of BD, in contrast to more recent research, which often includes a broader spectrum. Also, during the era when the diagnosis of BD was used primarily for patients with classical presentation, lithium was helpful for up to 80% of patients (Schou and Thomsen 1975), whereas in recent studies using broad criteria, the benefit of lithium has ranged from non-existent (Bowden et al. 2000) to 30% (Garnham et al. 2007). If the classical and broader spectrum type of BD were in some way contrasted in the contemporary studies, one would certainly find significant differences between these subgroups. For example, one could use the response to lithium prophylaxis as an approximation and a proxy for the classical manic-depressive illness, including in particular episodic course, lack of psychiatric comorbidity, and positive family history. Further supporting the differences between subtypes, offspring of lithium responder (LiR) parents with BD have been shown to be significantly more socially successful than those of lithium non-responder (LiNR) (e.g., Duffy et al. 2002).

In this paper we present findings related to an exploratory analysis of the association between SES and BD, divided on the basis of lithium response.

## Methods

Subjects were patients diagnosed with BDI or BDII between 1 January 1971 and 31 December 2012 and who participated in clinical and treatment research studies that took place at the Mood Disorder Programs in the Hamilton Psychiatric Hospital, McMaster University, and the Mood Disorders Centre of Ottawa. These individuals required stabilizing treatment and were given a sufficient therapeutic trial on lithium. All diagnoses were agreed upon by the research team, which included two or more research psychiatrists. Also, starting in 1978, the SADS-L interview was employed. Both programs, in Hamilton and in Ottawa, studied systematically the response to long-term stabilizing lithium treatment in all their bipolar patients. The resulting findings have been published and presented between 1970 and 2009 (Duffy et al. 2002).

Criteria used for lithium response were those applied in previous studies (e.g., Grof et al. 2009) and were also utilized in this analysis. In essence, patients classified as responders to lithium stabilization had, according to their course of illness, a marked risk of further recurrences. Yet on lithium treatment, these individuals remained free of recurrences for three or more years. LiNR patients experienced two or more recurrences despite adequate lithium treatment identified by sufficient serum lithium level at the time of recurrence (Garnham et al. 2007;Duffy et al. 2007;Turecki et al. 1998). All study participants were classified as either LiR or LiNR during their treatment, prior to the onset of this study, and no participants were excluded based on their response to lithium.

To avoid the distortion that could be involved in diagnosing subtypes of BD in retrospect, we decided to employ lithium response as a proxy for the diagnosis of the classical type of BD (manic-depressive illness). Individuals with good response to lithium prophylaxis have characteristics compatible with the description of the classic type of BD and create a relatively homogeneous subgroup (Alda 2004). Their clinical characteristics correspond to those of classical manic-depressive illness, including an episodic course, positive family history, and the absence of comorbidity. The advantage of using good response as a proxy for the diagnosis of the classical manic-depressive type of BD is that the identification of a responder depends primarily on the patient's behavior rather than on the clinician's interpretation of the bipolar subtype in retrospect.

Upon entry into treatment, SES was measured using the Hollingshead SES scale, which takes into account education and occupation. Education scores range from 1 (below grade 7) to 7 (graduate professional training). Occupation scores range from 1 (e.g., farm laborers and welfare recipients) to 9 (e.g., higher executives and major professionals). These numbers are weighed and calculated separately for each working spouse and then averaged between spouses, unless there is only one working spouse. The total number is assigned a final score, which represents social class ranking, ranging from 1 to 5, where 1 is the lowest class and 5 is the highest (Hollingshead 1975). The determination of SES is complicated in particular by the fact that the socioeconomic situation in the population has been evolving. The Hollingshead categories are based on United States Census data from the 1970s. Therefore, it has been criticized for using outdated categories of occupation. However, it has been widely used and most often in the studies whose discrepancies we attempt to interpret.

Combining the data from the programs in Hamilton and Ottawa was possible as both were directed by the same people and have had a similar orientation and data collection system. Furthermore, the combination reflected better the SES of the population. The Ottawa area, as the national capital, has a large proportion of blue-collar workers with a higher SES, whereas the Hamilton area is distinctly industrial.

Because of the nature of the variables, the relationship between SES and lithium response (and thus bipolar subtype) has been tested using the Mann–Whitney *U* test. The categorical variables were compared using Fisher's exact test. The testing of the influence of other variables on the relationship was approximated by Cox logistic regression.

Research studies in which SES and clinical information was collected and coded were approved by the corresponding research ethics boards. Data used in this study are fully anonymous.

## Results

This analysis included 652 individuals with BDI or BDII. Of these individuals, 260 patients came from McMaster Medical Center in Hamilton and 392 came from the Ottawa Mood Disorder Center. The Ottawa group did have a somewhat higher average SES value than the Hamilton group (Ottawa 3.25, standard deviation (SD) = 1.24; Hamilton 2.96, SD = 1.07; Tables 1 and 2).

**Table 1:** **SES information**

SES	Mean	SD
Centers/locations		
Hamilton	2.95	1.07
Ottawa	3.25	1.24
Gender		
Male	3.18	1.21
Female	2.11	1.17
Lithium response		
LiR	3.50	1.12
LiNR	2.94	1.17
Diagnosis		
BDI	3.24	1.21
BDII	3.09	1.17

**Table 2:** **Lithium response information**

Lithium response	LiR ***N*** (%)	LiNR ***N*** (%)
Gender		
Male (256)	95 (37.1)	161 (62.9)
Female (396)	133 (33.6)	263 (66.4)
Diagnosis		
BDI (209)	86 (41.1)	123 (58.9)
BDII (443)	142 (32.1)	301 (67.9)

The study sample consisted of 256 (39.3%) males and 396 (60.7%) females. The participants were classified as either LiR or LiNR, with 228 LiR individuals and 424 LiNR individuals. The mean SES for LiR individuals was higher at 3.50 (SD = 1.12) compared to 2.94 (SD = 1.17) for LiNR individuals. It is also interesting to note that the combined mean of the SES for both LiR and LiNR individuals was found to be 3.13 (SD = 1.19). Gender was also investigated to determine its effect on SES. A small difference was noted in that despite the fact that there were more females in the study compared to males, the males still demonstrated a higher mean SES compared to the females, with 3.18 (SD = 1.21) for the males and 2.11 (SD = 1.17) for the females.

The SES of the LiR patients was higher than that of the LiNR patients (Mann–Whitney *U* test, *p* < 0.001). The influence of other variables (BDI versus BDII, gender, and age) did not alter the significance of this relationship. A larger proportion of LiNR were BDII rather than BDI, but the relationship to SES did not reach statistical significance*.*

## Discussion

BD was once defined classically, as manic-depressive illness, but in recent years this definition has expanded to include bipolar spectrum disorders with diagnostic criteria far broader than used previously (Grof and Muller-Oerlinghausen 2009). Upon the inclusion of this broader category of BD (Healy 2006), the prevalence of BD rose from an estimated 0.6% of the population (Harris et al. 2005) to possibly more than 5% (Angst 1998). This very significant increase, potentially related to the change in diagnostic criteria, should be considered when comparing the statistical differences found in SES from older studies to more contemporary studies.

In order to determine whether this broadening of diagnostic criteria could be responsible for the shift in relative SES, a distinction first has to be made between the classical form of BD and the bipolar spectrum. There is evidence that patients who respond to lithium stabilization have primarily features of the classical manic-depressive illness, such as an episodically relapsing-remitting disease course, no psychiatric comorbidity, and a positive family history. Therefore, lithium responders can serve as a proxy for the classical manic-depressive type of BD.

This analysis revealed that in a contemporary sample, BD is associated with a significantly higher SES in the LiR group, compared to LiNR. These observations supported by past literature (Coryell et al. 1989;Lenzi et al. 1993;Tietze et al. 1941;Luxenburger 1933;Noreik and Odegaard 1966;Rao 1966;Gershon and Liebowitz 1975;Petterson 1977) suggest that those with the classical lithium-responsive form of BD are likely to have a relatively higher SES than their bipolar spectrum counterparts. Conversely, as the more recent literature has illustrated (Schoeyen et al. 2011;Tsuchiya et al. 2004;Goldstein et al. 2010), those with bipolar spectrum disorders non-responsive to lithium had a significantly lower SES (*p* = 0.001).

Assuming that SES and social achievements are correlated, this finding in adults appears consistent with an observation by Duffy et al. (2002) in bipolar offspring. In offspring of bipolar parents studied currently, Duffy et al. (2002) found distinctly higher social achievements in children of LiR (classical type of BD), as compared with children of LiNR. Presumably, those with a gifted premorbid development may go on to enjoy a higher SES, while those with problems in social and academic functioning later fall into lower SES groups.

These findings suggest that changes in diagnostic criteria may contribute to discrepancies in the literature regarding BD and SES. Specifically, earlier studies included patients with the classical diagnosis of manic-depressive illness whereas recent studies investigated patients diagnosed using a broader approach, reflecting the concept of bipolar spectrum. Early findings consistently showed an association between high SES and the diagnosis of BD (Malzberg 1956;Verdoux and Bourgeois 1995), whereas newer studies have failed to find any relationship at all (Schoeyen et al. 2011) and in some cases an opposite one (Tsuchiya et al. 2004). There have been speculations that these differences are due to methodological problems such as treatment bias, lack of appropriate reference samples, and failure to control for demographic variables (2011;Coryell et al. 1989). However, the differences between early and recent studies persist even when methodologies have not been dissimilar. This leaves room for other explanations as well.

Several factors could have contributed to the SES difference between LiR and LiNR. Stabilized patients are likely to function better, but we do not think that this factor played a role because the SES was determined at the time when patients entered the treatment. Furthermore, in general, people with a higher SES have better access to treatment and are often more likely to seek out help, factors which could certainly introduce a selection bias. For example, if people with higher SES are more likely to seek professional help, then this surely contributes to the prevalence of higher SES in BD individuals. Patients were admitted to the Mood Disorder Programs regardless of whether or not they were responsive to lithium, therefore minimizing, though not fully eliminating, systematic bias caused by access to care.

This study has several other limitations. One of them is that the SES scores were measured using the Hollingshead index, an outdated measure with some methodological shortcomings. Additionally, many studies that our results are being compared with used different methodological measures of SES. However, the Hollingshead index has been shown to correlate with other measures of social class, including more contemporary measures (Cirino et al. 2002). In addition, this index has been widely used in the psychiatric literature, which we are attempting to interpret here. The fact that our sample had a relatively high average SES could have been a reflection of either the inapplicability of the Hollingshead score or selection bias. Patients with BD recruited from the Mood Disorders Centre of Ottawa come primarily from the National Capital Area, which has one of the highest family incomes when compared to other urban communities in Canada (Finlayson 2011). In the USA, and likely other Western countries such as Canada, economic indicators are the most sensitive of SES and are independent of other socioeconomic variables such as education (Bauer et al. 2011). However, other countries may have different primary indicators of SES, such as family name, religion, or education.

Although there is evidence to suggest that lithium response can serve as a proxy for classical manic-depressive illness (Alda 2004), it is possible that this creates some selection bias since lithium had often been the first-line treatment for those with bipolar disorder. It is possible that some patients may have responded to other medication first, had it been administered.

Finally, another limitation to the study is that we did not find a suitable control population. The control population we do have comes exclusively from the National Capital Area and would not be suitable for comparison. A comparable control population would have been particularly helpful for addressing the generalizability of the findings.

## Conclusion

Our observation of socioeconomic differences between the LiR (classical manic-depressive) and LiNR (other subtypes) patients suggests that the discrepant findings regarding SES in BD could be in part explained by the change in diagnostic practices: early studies included mostly classical manic-depressive disorder while the patients in recent studies have been diagnosed according to much broader criteria. Future research should investigate SES differences by BD subtype and contrast with a comparison group to confirm these findings.

## Abbreviations

BD: Bipolar disorder
LiNR: Lithium non-responder
LiR: Lithium responder
SES: Socioeconomic status.
